# Supplementary figures and images for: Diurnal predators of restocked lab-reared and wild Diadema antillarum near artificial reefs in Saba
Source: PeerJ. 2023 Oct 12;11:e16189. doi: 10.7717/peerj.16189 (PMC10576963; doi:10.7717/peerj.16189)

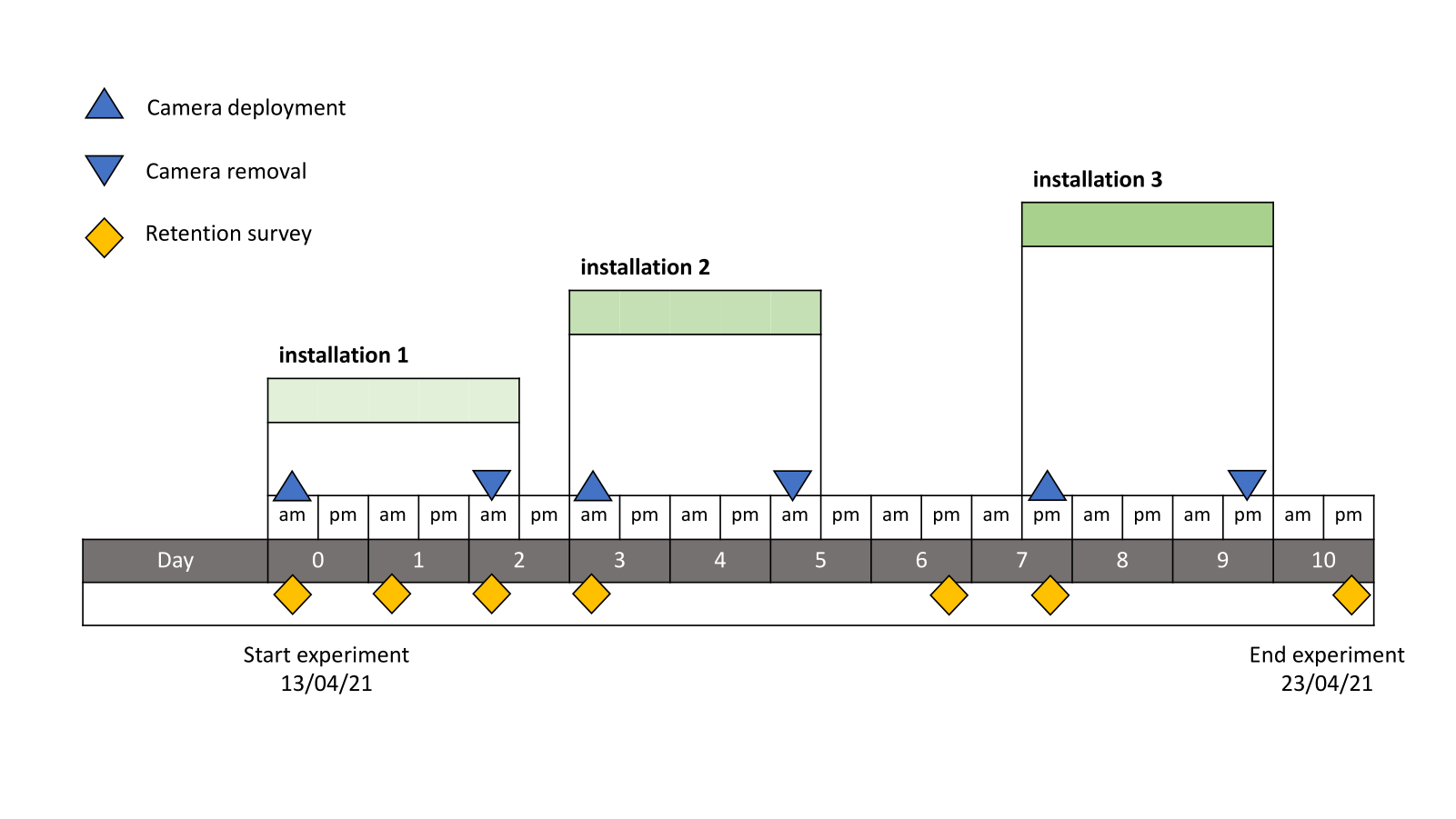

Supplement: Figure S1 — When camera deployments (blue triangles pointing up), camera retrievals (blue triangles pointing down), and retention counts (orange diamonds) were performed over the course of the experiment. [file peerj-11-16189-s002.png]
